# Gorilla in our midst: An online behavioral experiment builder

**DOI:** 10.3758/s13428-019-01237-x

**Published:** 2019-04-23

**Authors:** Alexander L. Anwyl-Irvine, Jessica Massonnié, Adam Flitton, Natasha Kirkham, Jo K. Evershed

**Affiliations:** 1 MRC Cognition and Brain Science Unit, University of Cambridge, Cambridge, UK; 2 Cauldron.sc: Cauldron Science, St Johns Innovation Centre, Cambridge, UK; 3 Centre for Brain and Cognitive Development, Birkbeck College, University of London, London, UK; 4 Human Behaviour and Cultural Evolution Group, University of Exeter, Exeter, UK

**Keywords:** Online methods, Remote testing, Browser timing, Attentional control, Online research, Timing accuracy

## Abstract

Behavioral researchers are increasingly conducting their studies online, to gain access to large and diverse samples that would be difficult to get in a laboratory environment. However, there are technical access barriers to building experiments online, and web browsers can present problems for consistent timing—an important issue with reaction-time-sensitive measures. For example, to ensure accuracy and test–retest reliability in presentation and response recording, experimenters need a working knowledge of programming languages such as JavaScript. We review some of the previous and current tools for online behavioral research, as well as how well they address the issues of usability and timing. We then present the Gorilla Experiment Builder (gorilla.sc), a fully tooled experiment authoring and deployment platform, designed to resolve many timing issues and make reliable online experimentation open and accessible to a wider range of technical abilities. To demonstrate the platform’s aptitude for accessible, reliable, and scalable research, we administered a task with a range of participant groups (primary school children and adults), settings (without supervision, at home, and under supervision, in both schools and public engagement events), equipment (participant’s own computer, computer supplied by the researcher), and connection types (personal internet connection, mobile phone 3G/4G). We used a simplified flanker task taken from the attentional network task (Rueda, Posner, & Rothbart, 2004). We replicated the “conflict network” effect in all these populations, demonstrating the platform’s capability to run reaction-time-sensitive experiments. Unresolved limitations of running experiments online are then discussed, along with potential solutions and some future features of the platform.

**Electronic supplementary material:**

The online version of this article (10.3758/s13428-019-01237-x) contains supplementary material, which is available to authorized users.

## Introduction

Behavioral research and experimental psychology are increasing their use of web browsers and the internet to reach larger (Adjerid & Kelley, 2018) and more diverse (Casler, Bickel, & Hackett, 2013) populations than has previously been feasible with lab-based methods. However, unique variables are introduced when working within an online environment. The experience of the user is the result of a large number of connected technologies, including the server (which hosts the experiment), the internet service provider (which delivers the data), the browser (which presents the experiment to the participant and measures their responses), and the content itself—which is determined by a mixture of media (e.g., audio/pictures/video) and code in different programming languages (e.g., JavaScript, HTML, CSS, PHP, Java). Linking these technologies is technically difficult, time-consuming, and costly. Consequently, until recently, online research was generally carried out—and scrutinized—by those with the resources to overcome these barriers.

The purpose of this article is threefold: first, to explore the problems inherent to running behavioral experiments online with web programming languages, the issues this can create for timing accuracy, and recent improvements that can mitigate these issues; second, to introduce Gorilla, an online experiment builder that uses best practices to overcome these timing issues and makes reliable online experimentation accessible and transparent to the majority of researchers; third, to demonstrate the timing accuracy and reliability provided by Gorilla. We achieved this last goal using data from a flanker task—which requires high timing fidelity—collected from a wide range of participants, settings, equipment, and internet connection types.

### JavaScript

The primary consideration for online experimenters in the present time is JavaScript, the language that is most commonly used to generate dynamic content on the web (such as an experiment). Its quirks (which are discussed later) can lead to problems with presentation time, and understanding it forms a large part of an access barrier.

JavaScript is at the more dynamic end of the programming language spectrum. It is weakly typed and allows core functionality to be easily modified. Weak typing means that variables do not have declared types; the user simply declares a variable and then uses it in their code. This is in contrast to strongly typed languages, in which the user must specify whether a variable they declare should be an integer, a string, or some other structure. This can lead to unnoticed idiosyncrasies—if a user writes code that attempts to divide a string by a number, or assign a number to a variable that was previously assigned to an array, JavaScript allows this to proceed. Similarly, JavaScript allows users to call functions without providing all the arguments to that function. This dynamic nature gives more flexibility, but at the cost of allowing mistakes or unintended consequences to creep in. By contrast, in a strongly typed language, incorrect assignments or missing function arguments would be marked as errors that the user should correct. This results in a more brittle, but safer, editing environment. JavaScript also allows a rare degree of modification of core structures—even the most fundamental building blocks (such as arrays) can have extra methods added to them. This can prove useful in some cases, but can easily create confusion as to which parts of the code are built-in and which parts are user defined. Together, these various factors create a programming environment that is very flexible, but one in which mistakes are easy to make and their consequences can go undetected by the designer (Richards, Lebresne, Burg, & Vitek, 2010). This is clearly not ideal for new users attempting to create controlled scientific experiments. Below we discuss two significant hurdles when building web experiments: inaccuracies in the timing of various experiment components in the browser, and the technical complexities involved in implementing an online study, including JavaScript’s contributions. These complexities present an access barrier to controlled online experiments for the average behavioral researcher.

### History of timing concerns

Timing concerns have been expressed regarding online studies (for an overview, see Woods, Velasco, Levitan, Wan, & Spence, 2015), and although many of these concerns are now historic for informed users—because solutions exist—they are still an issue for new users who may not be aware of them. These concerns can be divided into the timing of stimuli—that is, an image or sound is not presented for the duration you want—and the timing of response recording—that is, the participant did not press a button at the time they are recorded doing so. These inaccuracies have obvious implications for behavioral research, especially those using time-based measures such as reaction time (RT).

Several things might be driving these timing issues: First, in JavaScript programs, most processes within a single web-app or browser window pass through an event loop^1^—a single thread that decides what parts of the JavaScript code to run, and when. This loop comprises different types of queues. Queues that are managed synchronously wait until one task is complete before moving on. One example of a synchronously managed queue is the event queue, which stores an ordered list of things waiting to be run. Queues that are managed asynchronously will start new tasks instead of waiting for the preceding tasks to finish, such as the queue that manages loading resources (e.g., images). Most presentation changes are processed through the event loop in an asynchronous queue. This could be an animation frame updating, an image being rendered, or an object being dragged around. Variance in the order in which computations are in the queue, due to any experiment’s code competing with other code, can lead to inconsistent timing. When a synchronous call to the event loop requires a lot of time, it can “block” the loop—preventing everything else in the queue from passing through. For instance, you may try and present auditory and visual stimuli at the same time, but they could end up out of synchronization if blocking occurs—a common manifestation of this in web videos is unsynchronized audio and video.

Second, the computational load on the current browser window will slow the event loop down; variance in timing is, therefore, dependent on different computers, browsers, and computational loads (Jia, Guo, Wang, & Zhang, 2018). For a best-practices overview, see Garaizar and Reips (2018). Given the need for online research to make use of onsite computers such as those in homes or schools, the potential variance mentioned above is an important issue. A laptop with a single processor, a small amount of memory, and an out-of-date web browser is likely to struggle to present stimuli to the same accuracy as a multicore desktop with the most recent version of Google Chrome installed. These variances can represent variance of over 100 ms in presentation timing (Reimers & Stewart, 2016).

Third, by default, web browsers load external resources (such as images or videos) progressively as soon as the HTML elements that use them are added to the page. This results in the familiar effect of images “popping in” as the page loads incrementally. If each trial in an online task is treated as a normal web page, this “popping in” will lead to inaccurate timing. Clearly, such a variance in display times would be unsuitable for online research, but the effect can be mitigated by loading resources in advance. A direct solution is to simply load all the required resources, for all the trials, in advance of starting the task (Garaizar & Reips, 2018). This can be adequate for shorter tasks or tasks that use a small number of stimuli, but as the loading time increases, participants can become more likely to drop out, resulting in an increase in attrition.

The same concerns (with the exception of connection speed) can be applied to the recording of RTs, which are dependent on a JavaScript system called the “event system.” When a participant presses a mouse or keyboard button, recording of these responses (often through a piece of code called an “Event Listener”) gets added to the event loop. To give a concrete example, two computers could record different times of an identical mouse response based on their individual processing loads. It must be noted that this issue is *independent* of the browser receiving an event (such as a mouse click being polled by the operating system), for which there is a relatively fixed delay, which has been shown to be equivalent in nonbrowser software (de Leeuw & Motz, 2016)—this receiving delay is discussed later in the article. Timing of event recording using the browser system clock (which some JavaScript functions do) is also another source of variance—because different machines and operating systems will have different clock accuracies and update rates.

### Current state of the art

Presently, the improved processing capabilities in common browsers and computers, in concert with improvements in web-language standards—such as HTML5 and ECMAScript 6—offer the potential to overcome some concerns about presentation and response timings (Garaizar, Vadillo, & López-de Ipiña, 2012, 2014; Reimers & Stewart, 2015, 2016; Schmidt, 2001). This is because, in addition to standardized libraries (which improve the consistency of any potential web experiment between devices), these technologies use much more efficient interpreters, which are the elements of the browser that execute the code and implements computations. An example of this is Google’s V8, which improves processing speed—and therefore the speed of the event loop—significantly (Severance, 2012). In fact, several researchers have provided evidence that response times are comparable between browser-based applications and local applications (Barnhoorn, Haasnoot, Bocanegra, & van Steenbergen, 2015), even in poorly standardized domestic environments—that is, at home (Miller, Schmidt, Kirschbaum, & Enge, 2018).

A secondary benefit of recent browser improvements is scalability. If behavioral research continues to take advantage of the capacity for big data provided by the internet, it needs to produce scalable methods of data collection. Browsers are becoming more and more consistent in the technology they adopt—meaning that code will be interpreted more consistently across your experimental participants. At the time of writing, the standard for browser-based web apps is HTML5 (the World Wide Web Consortium, 2019, provides the current web standards) and the ECMAScript JavaScript (Zaytsev, 2019, shows that most browsers currently support ECMAScript 5 and above). ECMAScript (ES) is a set of standards that are implemented in JavaScript (but, can also be implemented in other environments—e.g., ActionScript in Flash), and browsers currently support a number of versions of this standard (see Zaytsev, 2019, for details). The combination of ES and HTML5, in addition to having improved timing, is also the most scalable. They reach the greatest number of users—with most browsers supporting them, which is in contrast with other technologies, such as Java plugins and Flash that are becoming inconsistently supported—in fact, Flash support has recently begun a departure from all major browsers.

### Access barriers

Often, to gain accurate timing and presentation, you must have a good understanding of key browser technologies. As in any application in computer science, there are multiple methods for achieving the same goal, and these may vary in the quality and reliability of the data they produce. One of the key resources for tutorials on web-based apps—the web itself—may lead users to use out-of-date or unsupported methods; with the fast-changing and exponentially expanding browser ecosystem, this is a problem for the average behavioral researcher (Ferdman, Minkov, Bekkerman, & Gefen, 2017). This level of complexity imposes an access barrier to creating a reliable web experiment—the researcher must have an understanding of the web ecosystem they operate in and know how to navigate its problems with appropriate tools.

However, tools are available that lower these barriers in various ways. Libraries, such as jsPsych (de Leeuw, 2015), give a toolbox of JavaScript commands that are implemented at a higher level of abstraction—therefore relieving the user of some implementation-level JavaScript knowledge. Hosting tools such as “Just Another Tool for Online Studies” (JATOS) allow users to host JavaScript and HTML studies (Lange, Kühn, & Filevich, 2015) and present the studies to their participants—this enables a research-specific server to be set up. However, with JATOS you still need to know how to set it up and manage your server, which requires a considerable level of technical knowledge. The user will also need to consider putting safeguards in place to manage unexpected server downtime caused by a whole range of issues. This may require setting up a back-up system or back-up server. A common issue is too many participants accessing the server at the same time, which can cause it to overload and likely prevent access to current users midexperiment—which can lead to data loss (Schmidt, 2000).

The solutions above function as “packaged software,” in which the user is responsible for all levels of implementation (i.e., browser, networking, hosting, data processing, legal compliance, regulatory compliance and insurance)—in the behavioral research use-case, this requires multiple tools to be stitched together (e.g., jsPsych in the browser and JATOS for hosting). This itself presents another access barrier, as the user then must understand—to some extent—details of the web server (e.g., how many concurrent connections their hosted experiment will be able to take), hosting (the download/upload speeds), the database (where and how data will be stored; e.g., in JavaScript object notation format, or in a relational database), and how the participants are accessing their experiment and how they are connected (e.g., through Prolific.ac or Mechanical Turk).

One way to lower these barriers is to provide a platform to manage all of this for the user, commonly known as *software as a service* (SaaS; Turner, Budgen, & Brereton, 2003). All of the above can be set up, monitored, and updated for the experimenter, while also providing as consistent and reproducible an environment as possible—something that is often a concern for web research. One recent example is the online implementation of PsyToolkit (Stoet, 2017), through which users can create, host, and run experiments on a managed web server and interface; however, there is still a requirement to write out the experiment in code, which represents another access limitation.

Some other tools exist in the space between SaaS and packaged software. PsychoPy3 (Peirce & MacAskill, 2018) is an open-source local application offering a graphical task builder and a Python programming library. It offers the ability to export experiments built in the task builder (but currently not those built using their Python library) to JavaScript, and then to a closed-source web platform based on GitLab (an repository -based b version control system) called Pavlovia.org, where users can host that particular task for data collection. Lab.js (Henninger, Mertens, Shevchenko, & Hilbig, 2017) is another task builder, which provides a web-based GUI, in which users can build a task and download a package containing the HTML, CSS, and JavaScript needed to run a study. Users are then able to export this for hosting on their own or on third-party servers. Neither of these tools functions fully as SaaS, since they do not offer a fully integrated platform that allows you to build, host, distribute tasks for, and manage complex experimental designs (e.g., a multiday training study) without programming, in the same environment. A full comparison of packaged software, libraries, and hosting solutions can be found in Table 1.

**Table 1:** Comparison of tools available for the collection of behavioral data, both online and offline

Type	Examples	$*	OS*	Description
Hosted experiment builder	Gorilla	$	CS	Gorilla contains a questionnaire builder, GUI task builder, Java Script code editor and an experiment design tool. Secure and reliable experiment hosting and data collection are part of the service provided. You can also host files from other task builders and libraries (i.e., jsPsych, Lab.js) that export to JavaScript with minor modification to connect to the Gorilla Server. Participants can be directed to an external resource (i.e., Qualtrics) and then return them to Gorilla.
Hosted survey tools	Qualtrics SurveyMonkey Lime Survey	$ $ $	CS CS OS	These allows users to collect questionnaire-type data and present media to participants. They are not designed for collecting reaction time data, for running behavioral science tasks or creating complex experimental designs.
Coding libraries	PsychoPy (Python) jsPsych (JavaScript) PsychToolBox (Matlab) PyGaze (Python)	F F F	OS OS OS	These help behavioral and neuroimaging researchers create tasks. These are built using programming languages. If web-compatible a server and database will be needed to host these online for data collection.
Task builders	E-Prime Presentation PsychoPy Builder Open Sesame PsyToolKit Lab.js	$ $ F F F F	CS CS OS OS OS OS	These are task creation tools. Many of these interface with neuroimaging equipment and eyetrackers. Some are more code based (i.e., PsyToolKit), whereas others provide pre-built tools (i.e., PsychoPy Builder). Some provide the ability to export JavaScript files (e.g., PsychoPy Builder and Lab.js) for online hosting via a 3rd party hosting solution. Free tools are often supported by community forums, whereas the paid solutions have help desks.
Hosted task builders	Inquisit Testable PsyToolKit on the web	$ $ F	CS CS OS	These are online task creation tools allowing you to build a task for use online, and also provide integrated hosting for that task. Some are more code based (i.e., Inquisit), whereas others are more tooled (i.e., Testable). The platform provides the hosting and data collection service for you.
Hosting solution	Pavlovia	F	CS	This is a grant funded and integrated hosting solution for PsychoPy Builder. You can also host files from other task builders and libraries that export to JavaScript.
Hosting libraries	JATOS TATOOL The Experiment Factory	F F F	OS OS OS	Hosting these libraries requires procuring and installing the source code on your own server that you may need to pay for. You will have to manage any updates to the library and implement any missing functionality that you need (e.g., integration with recruitment services). Additionally, you will need to maintain the server itself, and perform your own system administration, security and backups.

*Key: $, Paid for; F, Free to the user, often department or grant funded; OS, Open source; CS, Closed source

### The Gorilla Experiment Builder

Gorilla (www.gorilla.sc) is an online experiment builder whose aim is to lower the barrier to access, enabling all researchers and students to run online experiments (regardless of programming and networking knowledge). As well as giving greater access to web-based experiments, it reduces the risk of introducing higher noise in data (e.g., due to misuse of browser-based technology). By lowering the barrier, Gorilla aims to make online experiments available and transparent at all levels of ability. Currently, experiments have been conducted in Gorilla on a wide variety of topics, including cross-lingual priming (Poort & Rodd, 2017), the provision of lifestyle advice for cancer prevention (Usher-Smith et al., 2018), semantic variables and list memory (Pollock, 2018), narrative engagement (Richardson et al., 2018), trust and reputation in the sharing economy (Zloteanu, Harvey, Tuckett, & Livan, 2018), how individuals’ voice identities are formed (Lavan, Knight, & McGettigan, 2018), and auditory perception with degenerated music and speech (Jasmin, Dick, Holt, & Tierney, 2018). Also, several studies have preregistered reports, including explorations of object size and mental simulation of orientation (Chen, de Koning, & Zwaan, 2018) and the use of face regression models to study social perception (Jones, 2018). Additionally, Gorilla has also been mentioned in an article on the gamification of cognitive tests (Lumsden, Skinner, Coyle, Lawrence, & Munafò, 2017). Gorilla was launched in September 2016, and as of January 2019 over 5,000 users have signed up to Gorilla, across more than 400 academic institutions. In the last three months of 2018, data were collected from over 28,000 participants—an average of around 300 participants per day.

One of the greatest differences between Gorilla and the other tools mentioned above (a comprehensive comparison of these can be found in Table 1) is that it is an *experiment design tool*, not just a task-building or questionnaire tool. At the core of this is the Experiment Builder, a graphical tool that allows you to creatively reconfigure task and questionnaires into a wide number of different experiment designs without having to code. The interface is built around dragging and dropping nodes (which represent what the participant sees at that point, or modifications to their path through the experiment) and connecting them together with arrow lines. This modular approach makes it much easier for labs to reuse elements that have been created before, by themselves or by others. For instance, this allows any user to construct complex, counterbalanced, randomized, between-subjects designs with multiday delays and email reminders, with absolutely no programming needed. Examples of this can be seen in Table 2.

**Table 2:**
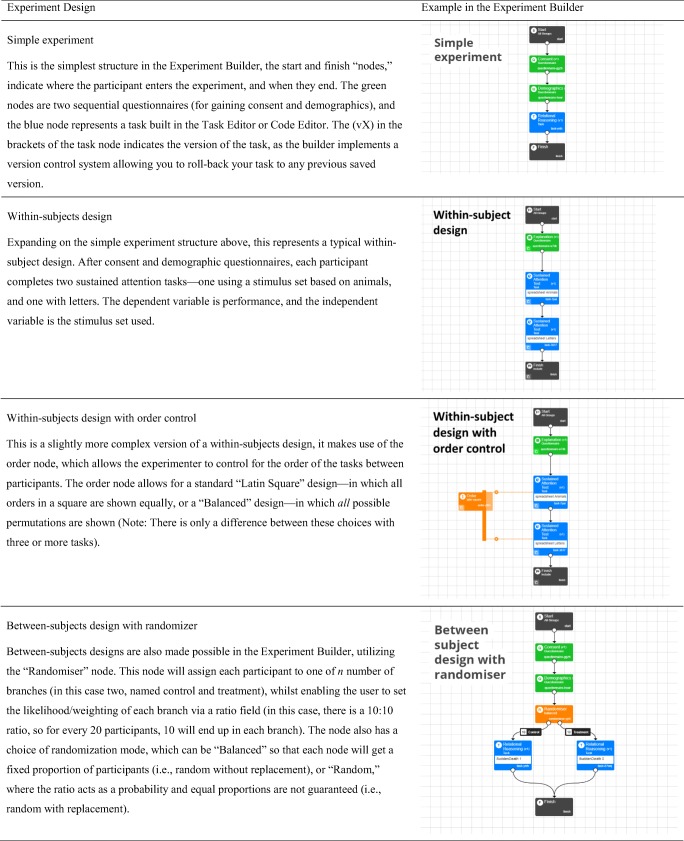

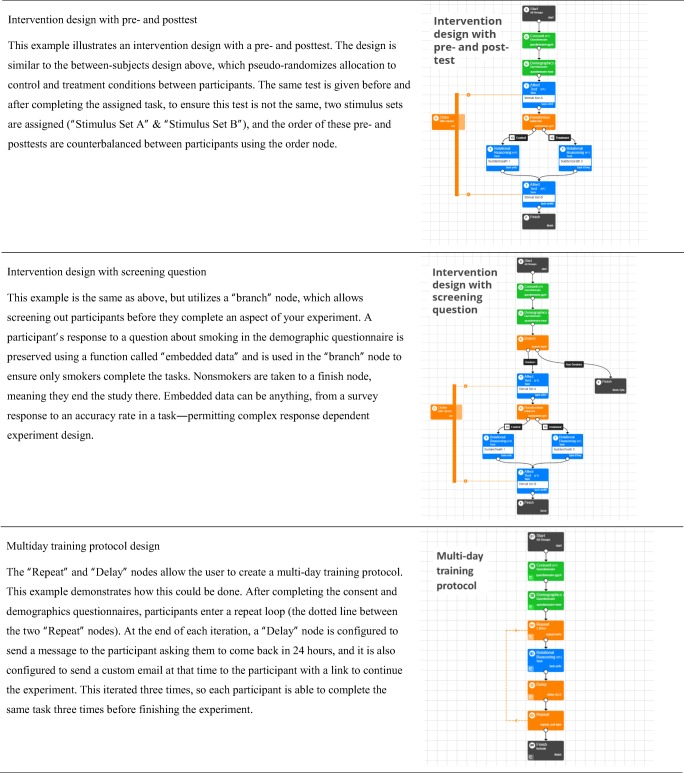
Examples of experimental designs possible to construct within Gorilla’s Experiment Builder interface

**Fig. 1:**
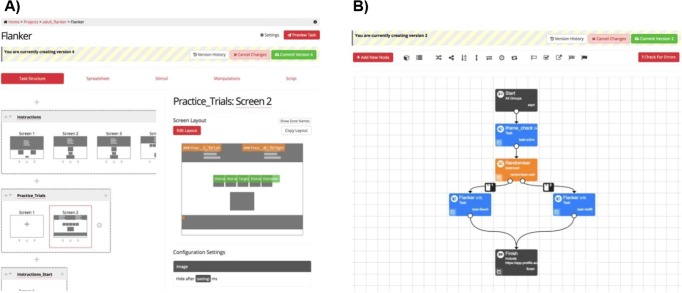
Example of the two main GUI elements of Gorilla. (**A**) The Task Builder, with a screen selected showing how a trial is laid out. (**B**) The Experiment Builder, showing a check for the participant, followed by a randomizer node that allocates the participant to one of two conditions, before sending them to a Finish node

Gorilla provides researchers with a managed environment in which to design, host, and run experiments. It is fully compliant with the EU General Data Protection Regulation and with NIHR and BPS guidelines, and it has backup communication methods for data in the event of server problems (to avoid data loss). A graphical user interface (GUI) is available for building questionnaires (called the “Questionnaire Builder”), experimental tasks (the “Task Builder”), and running the logic of experiments (“Experiment Builder”). For instance, a series of different attention and memory tasks could be constructed with the Task Builder, and their order of presentation would be controlled with the Experiment Builder. Both are fully implemented within a web browser and are illustrated in Fig. 1. This allows users with little or no programming experience to run online experiments, whilst controlling and monitoring presentation and response timing.

At the Experiment Builder level (Fig. 1B), users can create logic for the experiment through its nodes, which manage capabilities such as randomization, counterbalancing, branching, task switching, repeating, and delay functions. This range of functions makes it as easy to create longitudinal studies with complex behavior. An example could be a four-week training study with email reminders, in which participants would receive different tasks based on prior performance, or the experiment tree could just as easily enable a one-shot, between-subjects experiment. Additionally, Gorilla includes a redirect node that allows users to redirect participants to another hosted service and then send them back again. This allows users to use the powerful Experiment Builder functionality (i.e., multiday testing) while using a different service (such as Qualtrics) at the task or questionnaire level. Table 2 provides a more detailed explanation of several example experiments made in the builder.

The Task Builder (Fig. 1A) provides functionality at the task level. Each experimental task is separated into “displays” that are made of sequences of “screens.” Each screen can be configured by the user to contain an element of a trial, be that text, images, videos, audio, buttons, sliders, keyboard responses, progress bars, feedback, or a wide range of other stimuli and response options. See the full list here: https://gorilla.sc/support/articles/features. The content of these areas either can be static (such as instructions text) or can change on a per-trial basis (when the content is set using a spreadsheet). The presentation order of these screens is dependent on sequences defined in this same spreadsheet, in which blocked or complete randomization can take place on the trial level. Additionally, the Task Builder also has a “Script” tab, which allows the user to augment the functionality provided by Gorilla with JavaScript. This allows users to use the GUI and JavaScript side by side. There is also a separate “Code Editor,” which provides a developmental environment to make experiments purely in code. This allows users to include external libraries, such as jsPsych. The purpose of the Code Editor is to provide a secure and reliable service for hosting, data storage, and participant management for tasks written in code.

Using tools like the Code Editor, users can extend the functionality of Gorilla through use of the scripting tools, in which custom JavaScript commands, HTML templates, and an application programming interface (API) are available—an API is a set of functions that gives access to the platform’s functionality in the Code Editor, and also allows users to integrate third-party libraries into their experiments (e.g., tasks programmed in jsPsych). Therefore, Gorilla also can function as a learning platform through which users progress on to programming—while providing an API that manages more complex issues (such as timing and data management) that might cause a beginner to make errors. The Code Editor allows the inclusion of any external libraries (e.g., pixi.js for animation, OpenCV.js for image processing, or WebGazer.js for eyetracking). A full list of features is available at www.gorilla.sc/tools, and a tutorial is included in the supplementary materials.

### Timing control

A few techniques are utilized within Gorilla to control timing. To minimize any potential delays due to network speed (mentioned above), the resources from several trials are loaded in advance of presentation, a process called caching. Gorilla loads the assets required for the next few trials, begins the task, and then continues to load assets required for future trials while the participant completes the task. This strikes an optimal balance between ensuring that trials are ready to be displayed when they are reached, while preventing a lengthy load at the beginning of the task. This means that fluctuations in connection speed will not lead to erroneous presentation times. The presentation of stimuli are achieved using the requestAnimationFrame() function, which allows the software to count frames and run code when the screen is about to be refreshed, ensuring that screen-refreshing in the animation loop does not cause hugely inconsistent presentation. This method has previously been implemented to achieve accurate audio presentation (Reimers & Stewart, 2016) and accurate visual presentation (Yung, Cardoso-Leite, Dale, Bavelier, & Green, 2015). Rather than assuming that each frame is going to be presented for 16.667 ms, and presenting a stimulus for the nearest number of frames (something that commonly happens), Gorilla times each frame’s actual duration—using requestAnimationFrame(). The number of frames a stimulus is presented for can, therefore, be adjusted depending on the duration of each frame—so that most of the time a longer frame refresh (due to lag) will not lead to a longer stimulus duration. This method was used in the (now defunct) QRTEngine (Barnhoorn et al., 2015), and to our knowledge is not used in other experiment builders (for a detailed discussion of this particular issue, see the following GitHub issue, www.github.com/jspsych/jsPsych/issues/75, and the following blog post on the QRTEngine’s website, www.qrtengine.com/comparing-qrtengine-and-jspsych/).

RT is measured and presentation time is recorded using the performance.now() function, which is independent of the browser’s system clock, and therefore not impacted by changes to this over time. This is the same method used by QRTEngine, validated using a photodiode (Barnhoorn et al., 2015). Although performance.now() and its associated high-resolution timestamps offer the greatest accuracy, resolution has been reduced intentionally by all major browsers, in order to mitigate certain security threats (Kocher et al., 2018; Schwarz, Maurice, Gruss, & Mangard, 2017). In most browsers, the adjusted resolution is rounded to the nearest 1–5 ms, with 1 ms being the most common value (Mozilla, 2019). This is unlikely to be a permanent change, and will be improved when the vulnerabilities are better understood (Mozilla, 2019; Ritter & Mozilla, 2018).

Additionally, to maximize data quality, the user can restrict through the GUI which devices, browsers, and connection speeds participants will be allowed to have, and all these data are then recorded. This method allows for restriction of the participant’s environment, where only modern browser/device combinations are permitted, so that the above techniques—and timing accuracy—are enforced. The user is able to make their own call, in a trade-off between potential populations of participants and restrictions on them to promote accurate timing, dependent on the particulars of the task or study.

### Case study

As a case study, a flanker experiment was chosen to illustrate the platform’s capability for accurate presentation and response timing. To demonstrate Gorilla’s ability to work within varied setups, different participant groups (primary school children and adults in both the UK and France), settings (without supervision, at home, and under supervision, in schools and in public engagement events), equipment (own computers, computer supplied by researcher), and connection types (personal internet connection, mobile phone 3G/4G) were selected.

We ran a simplified flanker task taken from the attentional network task (ANT; Fan, McCandliss, Sommer, Raz, & Posner, 2002; Rueda, Posner, & Rothbart, 2004). This task measures attentional skills, following attentional network theory. In the original ANT studies, three attentional networks were characterized: alerting (a global increase in attention, delimited in time but not in space), orienting (the capacity to spatially shift attention to an external cue), and executive control (the resolution of conflicts between different stimuli). For the purpose of this article, and for the sake of simplicity, we will focus on the executive control component. This contrast was chosen because MacLeod et al. (2010) found that it was highly powered and reliable, relative to the other conditions in the ANT. Participants responded as quickly as possible to a central stimulus that was pointing either in the same direction as identical flanking stimuli or in the opposite direction. Thus, there were both congruent (same direction) and incongruent (opposite direction) trials.

Research with this paradigm has robustly shows that RTs to congruent trials are faster than those to incongruent trials—Rueda et al. (2004) have termed this the “conflict network.” This RT difference, although significant, is often less that 100 ms, and thus very accurately timed visual presentation and accurate recording of responses are necessary. Crump, McDonnell, and Gureckis (2013) successfully replicated the results of a similar flanker task online, using Amazon Mechanical Turk, with letters as the targets and flankers, so we know this can be an RT-sensitive task that works online. Crump et al. coded this task in JavaScript and HTML and managed the hosting and data storage themselves; however, the present versions of the experiment were created and run entirely using Gorilla’s GUI. We hypothesized that the previously recorded conflict RT difference would be replicated on this platform.

## Experiment 1

### Method

#### Participants

Data were drawn from three independent groups. Group A was in Corsica, France, across six different primary classrooms. Group B was in three primary schools in London, UK. Group C was at a public engagement event carried out at a university in London.

In total, 270 elementary school children were recruited. Two participants were excluded for not performing above chance (< 60% accuracy) in the task. The final sample included 268 children (53.7% of females), between 4.38 and 12.14 years of age (*M* = 9.21, *SD* = 1.58). Details about the demographics for each group are provided in Table 3. Informed written parental consent was obtained for each participant, in accordance with the university’s Ethics Committee.

**Table 3:** Sample size, age, and gender of the participants for each of the three groups

	Size	Gender (% female)	Age			
			Min	Max	Mean	*SD*
Group A	116	49.1	7.98	11.38	9.95	0.69
Group B	43	60.5	8.82	11.19	9.85	0.55
Group C	109	56.0	4.38	12.14	8.18	1.93

Age range is represented by the Min and Max columns. Group A was children in school in Corsica, France, Group B consisted of children in schools in London, UK, Group C consisted of children attending a university public engagement event in London

#### Procedure

In all three groups, participants were tested in individual sessions, supervised by a trained experimenter. Although great care was taken to perform the task in a quiet place, noise from adjacent rooms sometimes occurred in the school groups (A and B). To prevent children from getting distracted, they were provided with noise-cancelling headphones (noise reduction rating of 34dB; ANSI S3.19 and CE EN352-1 approved).

The task was carried out using the Safari web browser on a Mac OS X operating system. Because a stable internet connection was often lacking in schools, in Groups A and B, a mobile-phone internet connection was used—this could vary from 3G to 4G.

#### Flanker task

**Fig. 2:**
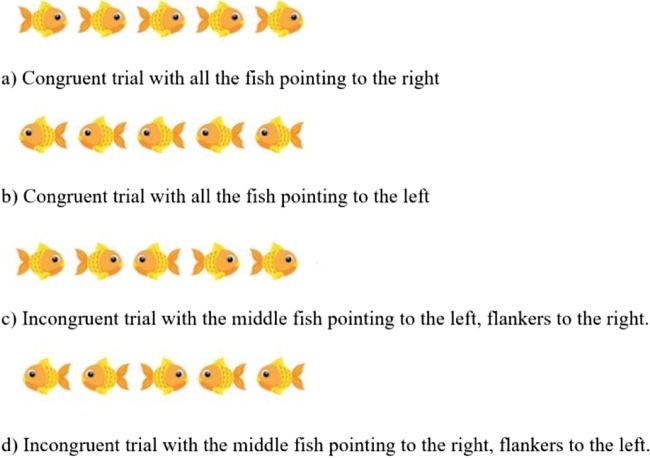
Trial types for Experiment 1: Different conditions used in the flanker task

The flanker task was adapted from that created by Rueda et al. (2004). A horizontal row of five cartoon fish were presented in the center of the screen (see Fig. 2), and participants had to indicate the direction the middle fish was pointing (either to the left, or right), by pressing the “X” or “M” buttons on the keyboard. These buttons were selected so that children could put one hand on each response key. Buttons were covered by arrows stickers (left arrow for “X”; right arrow for “M”) to avoid memory load. The task has two trial types: *congruent* and *incongruent*. In *congruent* trials, the middle fish was pointing in the same direction as the flanking fish. In the *incongruent* trials, the middle fish was pointing in the opposite direction. Participants were asked to answer as quickly and accurately as possible. The materials used in this experiment can be previewed and cloned on *Gorilla Open Materials* at https://gorilla.sc/openmaterials/36172.

After the experimenter had introduced the task, there were 12 practice trials, with immediate feedback on the screen. A red cross was displayed if children answered incorrectly, and a green tick was shown if they answered correctly. Instructions were clarified by the experimenter if necessary. After the practice trials, four blocks of 24 trials each were presented. Self-paced breaks were provided between the blocks. For each participant, 50% of the trials were congruent, and the direction of the middle fish varied randomly between left and right. Four types of trials were therefore presented (see Fig. 2): all the fish pointing to the right (25%), all the fish pointing to the left (25%), middle fish pointing to the right and flanking fish to the left (25%), and middle fish pointing to the left and flanking fish to the right (25%).

**Fig. 3:**
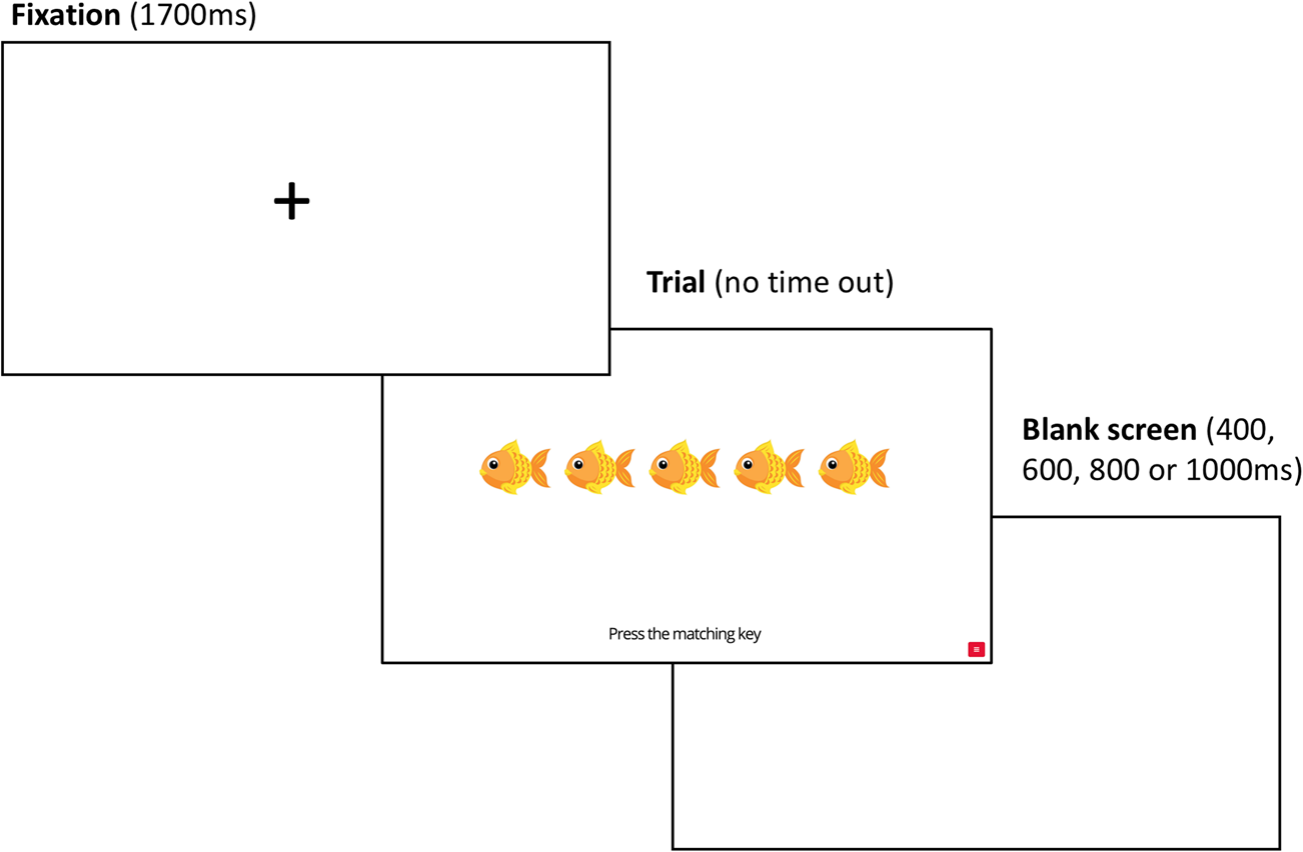
Time course of a typical trial in Experiment 1. These screens represent what the participant was seeing within the web browser

As is shown in Fig. 3, for each trial, a fixation cross was displayed for 1,700 ms. The cross was followed by the presentation of the fish stimuli, which stayed on screen until a valid response (either “X” or “M”) was provided. A blank screen was then displayed before the next trial. The duration of the blank screen varied randomly between 400, 600, 800, and 1,000 ms. Overall, the task took no more than 10 min.

#### Power calculations

The main flanker effect reported in Rueda et al.’s (2004) ANT analysis of variance (ANOVA) results (Exp. 1) was *F*(2, 88) = 61.92, *p* < .001. They did not report the effect size, so this permits us only to estimate the effect size using partial eta squared. This was calculated using the calculator provided by Lakens (2013), as *η*_p_^2^ = .58 (95% CI = .44–.67). Using G*Power (Faul, Erdfelder, Buchner, & Lang, 2009), an a priori power calculation was computed for a mixed-factor analysis of covariance (ANCOVA) with three groups, and a measurement correlation (congruent*incongruent) of .81 (taken from internal correlation of this measure reported in MacLeod et al., 2010). To reach a power above .95, a sample of 15 would be needed for each of our groups—we included in excess of this number, to increase sensitivity and provide power of > .99.

### Results

#### Data preprocessing

RTs for correct answers were computed. RTs under 200 ms were excluded, since they were too short to follow the perception and generation of response to the stimulus, and therefore are likely to be the result of anticipatory guesses, and do not relate to the process of interest (Whelan, 2008; see also the studies on visual awareness from Koivisto & Grassini, 2016, and Rutiku, Aru, & Bachmann, 2016).

Furthermore, RTs more than three standard deviations from the mean of each participant were excluded, in order to prevent extreme values from influencing the results (in some instances, children were asking a question in the middle of the trial; Whelan, 2008).

The accuracy score (number of correct answers/total number of trials) was calculated after trials were excluded for having RTs greater than three standard deviations from the mean, and/or less than 200 ms.

#### Accuracy

A mixed-factor ANCOVA was performed, with congruency as a within-subjects factor (two levels: accuracy for congruent trials, accuracy for incongruent trials), group as a between-subjects factor (three levels: Group A, Group B, Group C), and age as a covariate. We found a significant main effect of congruency on participants’ accuracy [*F*(1, 264) = 9.02, *p* = .003, *η*_p_^2^ = .033]. Although performance was at ceiling for both types of trials, participants were more accurate for congruent than for incongruent trials (see Table 4). This effect significantly interacted with participants’ age, *F*(1, 264) = 6.80, *p* = .010, *η*_p_^2^ = .025], but not with participants’ group [*F*(2, 264) = .501, *p* = .607, *η*_p_^2^ = .004]. To shed light on this interaction effect, the difference in accuracy scores between congruent trials and incongruent trials was computed for each participant. This difference diminished with age (*r* = – .22, *p* < .001).

**Table 4:** Accuracy and reaction times of participants, averaged (mean) over all groups, split by congruency

	Accuracy (%)	RT (ms)
Congruent	97.79 (0.18)	887.79 (17.10)
Incongruent	96.88 (0.31)	950.12 (23.71)

Standard errors of the means are shown in parentheses

**Fig. 4:**
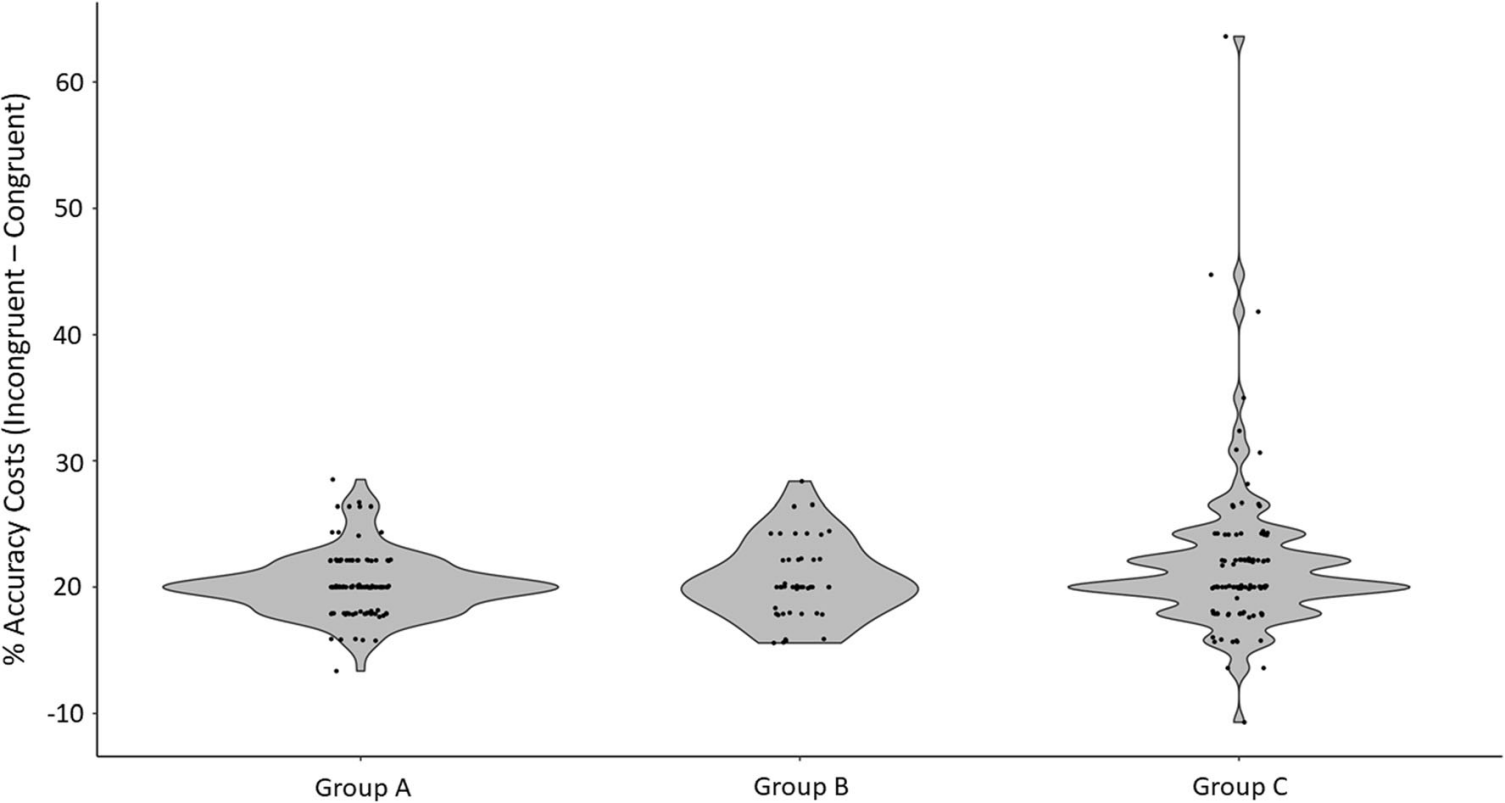
Distribution of accuracy differences between congruent and incongruent trials, for each group in Experiment 1. Group A was children in school in Corsica, France; Group B consisted of children in schools in London, UK; and Group C consisted of children attending a university public engagement event in London

The results from the ANCOVA should, however, be interpreted with caution, since two assumptions were violated in the present data. First, the distributions of accuracy scores in each of the three groups were skewed and did not follow a normal distribution (for Group A, Shapiro–Wilk *W* = .896, *p* < .001; for Group B, *W* = .943, *p* = .034; for Group C, *W* = .694, *p* < .001). Second, Levene’s test for equality of variances between groups was significant [for congruent trials: *F*(2, 265) = 5.75, *p* = .004; for incongruent trials: *F*(2, 265) = 13.90, *p* < .001]. The distribution of the data is represented in Fig. 4.

Due to these violations, the nonparametric Friedman test was carried out, which is tolerant of nonnormality. It also revealed a significant effect of congruency on accuracy scores [*χ*^2^(1) = 5.17, *p* < .023]. Further nonparametric tests were also carried out, to test whether the congruency effects differed between the three groups of participants. A Welch test for independent samples, tolerant of the nonequal variances between groups, indicated that the differences in accuracy between congruent and incongruent trials were not similar across groups [*F*(2, 108.53) = 3.25, *p* = .042]. Games–Howell post-hoc comparisons indicated that this effect was driven by the difference between Group A and Group C (*p* = .032). Groups A and B did not significantly differ from each other (*p* = .80), and neither did Groups B and C (*p* = .21). Descriptive statistics are reported in Table 5.

**Table 5:** Average differences in accuracy between congruent and incongruent trials, per participants’ group

	Accuracy difference (accuracy congruent – accuracy incongruent)
Group A	0.18 (0.23)
Group B	0.52 (0.47)
Group C	1.82 (0.60)

Standard errors of the means are shown in parentheses

However, as we reported in Table 3, the participants in Group C were younger than those in Group A, and the difference in accuracy between congruent and incongruent trials is generally larger for younger children. To check whether the group differences revealed by the Welch test were driven by this age difference, the “accuracy difference” scores (congruent – incongruent accuracy) were regressed on age, and the Welch test was performed on the residuals. The difference between participants’ groups was then nonsignificant [*F*(2, 108.48) = .396, *p* = .674], indicating that the previous Welch test results were likely driven by age.

#### Reaction time

A mixed-factor ANCOVA was performed, with congruency as a within-subjects factor (two levels: RT for congruent trials, RT for incongruent trials), group as a between-subjects factor (three levels: Group A, Group B, Group C), and age as a covariate. We found a main effect of congruency on participants’ RTs [*F*(1, 264) = 18.92, *p* < .001, *η*_p_^2^ = .067]. Participants took longer to provide the correct answers for incongruent than for congruent trials (see Table 4). This effect significantly interacted with age, *F*(1, 264) = 11.36, *p* = .001, *η*_p_^2^ = .041], but not with group type [*F*(2, 264) = .594, *p* = .553, *η*_p_^2^ = .004]. To better understand this interaction effect, RTs costs were calculated by subtracting the mean RTs to the congruent trials from the mean RTs to incongruent trials. Higher values indicate poorer inhibitory control, in that it took longer to give the correct answer for incongruent trials. RT costs decreased with age, indicating an improvement in inhibitory control over development (*r* = – .20, *p* = .001).

**Fig. 5:**
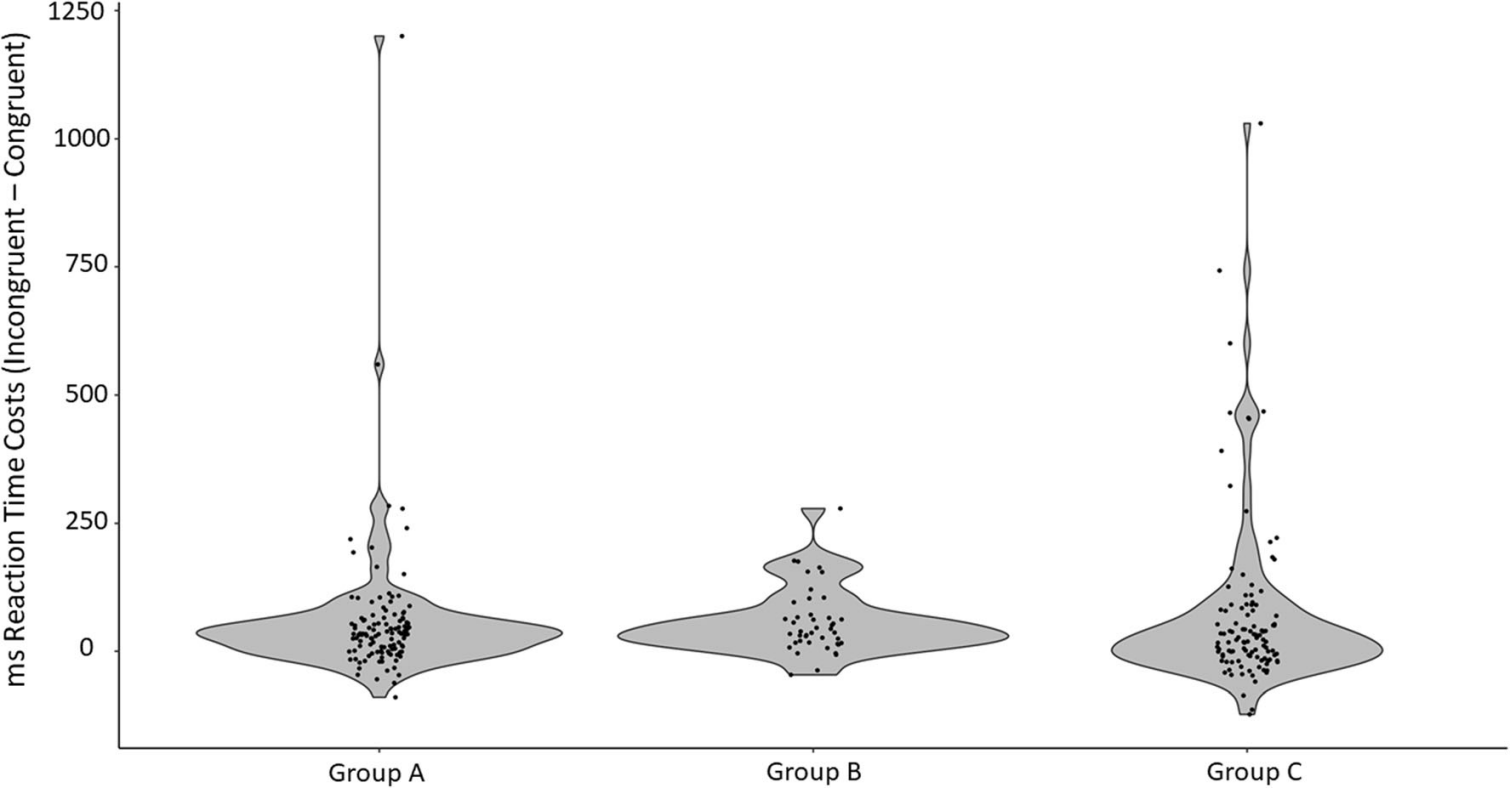
Distribution of RT differences between congruent and incongruent trials for each group in Experiment 1

Similarly to the analyses for accuracy scores, the RTs in each of the three groups were skewed and do not follow a normal distribution (for Group A, Shapiro–Wilk *W* = .476, *p* < .001; for Group B, *W* = .888, *p* = .034; for Group C, *W* = .649, *p* < .001). Second, Levene’s test for equality of variances between groups was significant [for congruent trials, *F*(2, 265) = 9.36, *p* < .001; for incongruent trials, *F*(2, 265) = 7.28, *p* < .001]. The distribution of the data is represented in Fig. 5. The nonparametric Friedman test, which is tolerant of nonnormal data, also revealed a significant effect of congruency on RTs for correct answers [*χ*^2^(1) = 55.37, *p* < .001]. A nonparametric Welch test for independent samples—tolerant of the nonequal distributions between groups—was carried out, indicating that RT costs (difference between congruent and incongruent trials) did not differ significantly between the three groups of participants [*F*(2, 165.22) = 0.335, *p* = .716], indicating that the main effect in the ANCOVA was unlikely to be driven by the group’s differences.

### Discussion

The flanker effect was successfully replicated in a sample of 268 children tested using Gorilla. This characterized the “conflict network,” with children taking longer to provide correct answers to incongruent than to congruent trials. This effect was lower than 100 ms (being 62.33 ms, on average). As we mentioned in the introduction, this small magnitude of difference requires both accurate visual timing and response recording to detect. Crucially, there was no interaction between the flanker effect and participants’ groups, even though their testing conditions differed greatly: Two groups were taken from schools, over a mobile-phone internet connection, and the third group was taken from a university setting, over a communal internet connection. In the case of accuracy, we did find a group difference in running nonparametric tests; however, it was shown that after accounting for the age difference between groups, this disappeared—which suggests this was not caused by the testing environment.

In each group, however, the pupils were supervised by a trained experimenter who guided them through the task and checked the quality of the internet connection. One of the potential benefits of web-based research is in reaching participants in various places (e.g., their own house), allowing for broad and unsupervised testing. Therefore, in Experiment 2 we tested whether the flanker effect would hold under such conditions, recruiting adult participants over Prolific and without supervision.

## Experiment 2

### Method

#### Participants

A total of 104 adults were recruited, five participants were excluded for not performing above chance (< 60% accuracy) in the task (these individuals also had accuracy in excess of three standard deviations from the mean). This left a sample of 99 adults (57.57% female), with a mean age of 30.32 years (*SD* = 6.64), ranging from 19 to 40 years old.

All participants were recruited online, through the Prolific.ac website, which allows the recruitment and administration of online tasks and questionnaires (Palan & Schitter, 2018). All participants were based in the United Kingdom and indicated normal or corrected-to-normal vision, English as a first language, and no history of mental illness or cognitive impairment. This experiment was conducted in line with Cauldron Science’s ethics code—which complies with the Declaration of Helsinki (World Medical Association, 2013). Informed consent was obtained through an online form, participants were informed they could opt out during the experiment without loss of payment.

Compensation for the task was £0.60 GBP, which on average translated to a rate of £8.70 per hour, as participants took an average of 4 min 8.36 s to complete the task.

In addition, the software recorded the operating system, web browser, and browser viewpoint size (the number of pixels that were displayed in the browser) of the users. The breakdown of these characteristics is shown in Tables 6 and 7.

**Table 6:** Breakdown of browsers and operating systems within the sample

	Count (Percentage)
Browser	
Chrome	75 (75.76%)
Safari	9 (9.09%)
Firefox	9 (9.09%)
Edge	3 (3.03%)
Other	3 (3.03%)
Operating system	
Windows 10	57 (57.58%)
Windows 7	17 (17.17%)
macOS	16 (16.16%)
Chromium	5 (5.05%)
Windows 8	4 (4.04%)

Total percentages of the sample are included in parentheses

**Table 7:** Viewport characteristics of the adult sample’s web browsers

	Mean (Pixels)	Std. deviation (Pixels)	Range (Pixels)
Horizontal	1,496.13	218.85	1,051–1,920
Vertical	759.40	141.66	582–1,266

The viewport is the area of a browser containing the information from a site

#### Procedure

Participants completed the task on their own computers at home and were not permitted to access the task on a tablet or smartphone. Before starting the task, participants read a description and instructions for taking part in the study, which asked them to open the experiment in a new window and note that the task would take around 5 min to complete (with an upper limit of 10 min). When the participants had consented to take part in the study on Prolific.ac, they were given a personalized link to the Gorilla website, in which the experimental task was presented. First, a check was loaded to ensure they had not opened the task embedded in the Prolific website (an option that was available at the time of writing), which would minimize distraction. Then the main section was administered in the browser; on completion of this they returned to Prolific.ac with a link including a verification code to receive payment.

#### Flanker task

**Fig. 6:**
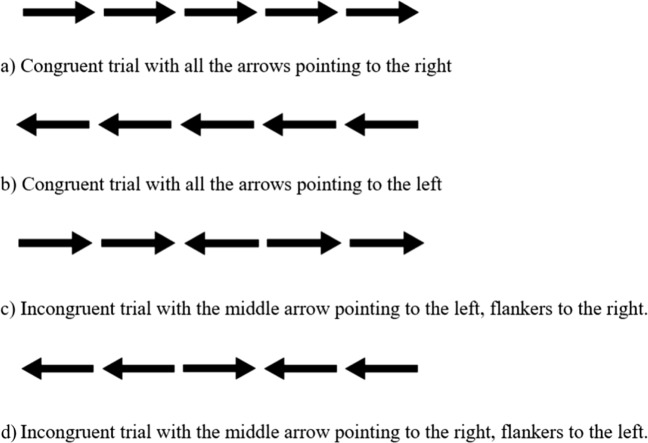
Trial types for Experiment 2: Different conditions used in the flanker task

**Fig. 7:**
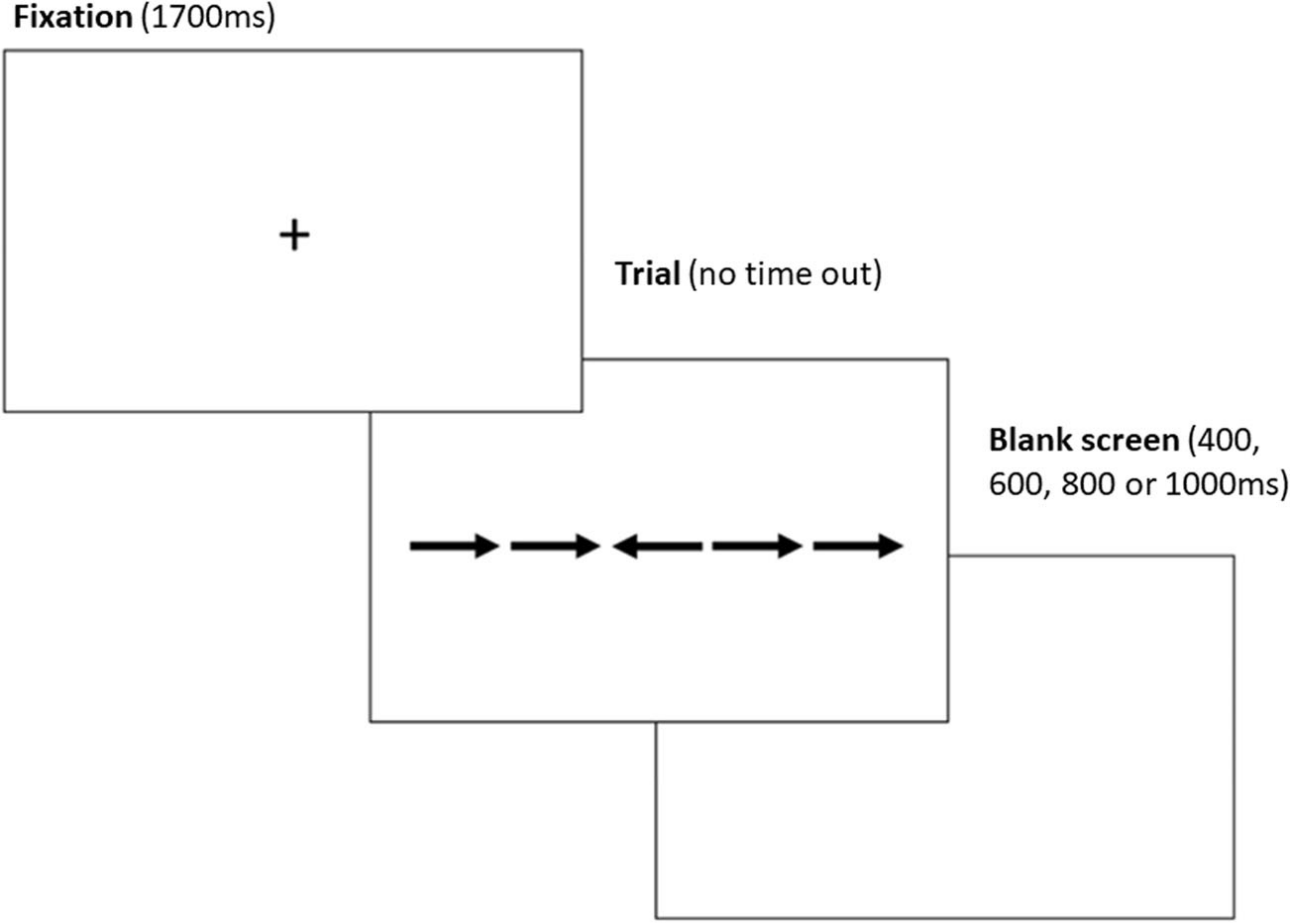
Time course of a typical trial in Experiment 2. These screens represent what the participant was seeing within the web browser

An adult version of the “conflict network” flanker task, adapted from the ANT used by Rueda et al. (2004). The mechanics, trial numbers, and conditions of this task were identical to those in Experiment 1; however, the stimuli were altered. The fish were replaced with arrows, as is typically done in adult studies (Fan et al., 2002; see Rueda et al., 2004, for a comparison of the child and adult versions). This is illustrated in Fig. 6, and the time course is illustrated in Fig. 7. The materials used in this experiment can be previewed and cloned on Gorilla Open Materials at www.gorilla.sc/openmaterials/36172.

Similarly to the children in Experiment 1, the adults were given written instructions and then completed 12 practice trials with immediate feedback. They moved on to complete four blocks of 24 trials (25% congruent–left, 25% congruent–right, 25% incongruent–left, 25% incongruent–right).

#### Power calculations

The main flanker effect reported in Rueda et al.’s (2004) adult arrow ANOVA results (Exp. 3) was *F*(2, 44) = 142.82, *p* = *.*0019. They did not report the effect size, so this permitted us only to estimate the effect size using partial eta-squared. This was calculated using the calculator provided by Lakens (2013), as *η*_p_^2^ = .87 (95% CI: .78–.90).

However, since our planned comparisons for this group were simple (a *t* test for mean RT and accuracy for incongruent vs. congruent trials), we calculated power using the reported mean and standard deviation values from Fan et al. (2002); Rueda et al. (2004) did not report the standard deviation, so this was not possible using their data. The mean RTs were 530 ms (*SD* = 49) for congruent trials and 605 ms (*SD* = 59) for incongruent trials. Using an a priori calculation from the G*Power software, this gave us a calculated effect size of *d* = 1.38 and a sample size of 26 to reach a power of .96. However, this assumed that we were working in a comparable environment, which was not the case, due to increased potential noise. Our sample size was therefore much larger than the original article to account for increased noise, giving us a calculated power of > .99.

### Results

#### Data preprocessing

As in Experiment 1, trials with RTs more than three standard deviations from the mean and/or less than 200 ms were excluded from both the accuracy and RT analyses.

#### Accuracy

The accuracy scores were computed over the total number of trials for each condition (congruent and incongruent). These means are shown in Table 8. As we mentioned above, five participants were excluded for accuracy scores that were not above chance. Accuracy was distributed nonnormally (Shapiro–Wilk *W* = .819, *p* < .001), so a Wilcoxon signed-rank test was used to compare the mean accuracies across the two types of trials. This provided evidence for a significant difference between the two means (1.72% difference, *W* = 1,242, *p* < .001) with a rank-biserial correlation of *r*_rb_ = .49 (an estimation of effect size for nonparametric data; Hentschke & Stüttgen, 2011).

**Table 8:** Average accuracy and correct trials reaction times for congruent and incongruent trials

	Accuracy (%)	RT (ms)
Congruent	99.28 (0.11)	498.72 (9.38)
Incongruent	97.56 (0.33)	527.81 (10.80)

Standard errors are in parentheses

#### Reaction time

The average RT was calculated for the two trial types—congruent and incongruent. Means and standard errors are reported in Table 8. RTs were only calculated for correct trials, since the accuracy rates were at ceiling. As above, the Shapiro–Wilk test suggested that the data were distributed nonnormally (*W* = .748 *p* < .001), so a Wilcoxon signed-rank test was used to compare the differences in mean RTs. This test suggested a significant difference between the two means (29.1-ms difference, *W* = .414, *p* < .001) with a rank-biserial correlation of *r*_rb_ = .83.

### Discussion

The “conflict network” effect was observed and replicated. This was encouraging, given the decrease in signal to noise that variance in operating system, web-browser, and screen size (shown above) would contribute toward this type of task. However, the effect of 29.1 ms was smaller than that observed in the original lab-based study (120 ms), and still smaller than the average effect of 109 ms reported in a meta-analysis of lab studies by MacLeod et al. (2010). This is likely due to variance in a remote environment, which may not be surprising, as MacLeod et al. (2010) found that there was large variance in the RT differences (congruent vs. incongruent) between and within participants over multiple studies—1,655 and 305 ms, respectively. Our smaller observed difference is also potentially driven by reduced RT variance. The average standard error in Experiment 1 was 20 ms, whereas it was around 10 ms in Experiment 2—possibly leading to the lower than expected difference in RT. We are unable to compare our variance with the original article’s child ANT results, as standard error or deviations were not reported. As a nearest online comparison, Crump et al.’s (2013) letter flankers’ difference between congruent and incongruent trials was 70 ms, which is closer to our observed difference, suggesting that online studies tend to find a smaller RT difference; however, the stimuli and task structure differed significantly between our implementation and Crump et al.’s.

One potential explanation for the faster RTs and decreased variance in the Prolific sample that we tested could be their unique setting—the framing and task goals of these participants were different from those of typical volunteers. Research investigating users on the Amazon Mechanical Turk platform found that they were more attentive than panel participants (Hauser & Schwarz, 2016), suggesting that internet populations are measurably different in their responses. Increased attentiveness could potentially lead to less within-subjects variance—this might be an avenue of research for a future study.

## General discussion

Gorrilla.sc is an experiment builder: a platform for the creation and administration of online behavioral experiments. It goes beyond an API, toolbox, or JavaScript engine and provides a full interface for task design and administration of experiments. It manages presentation time and response recording for the user, building on previous advances in browser-based research software without the requirement for programming or browser technology understanding. Utilizing these tools, measurement of the “conflict network” was successfully replicated online. The replication persisted across several different groups, children in primary schools in two countries, children at a public engagement event, and adults taking part on their own machines at home. This demonstrates that tasks built using this platform can be used in a wide range of situations—which have the potential to introduce unwanted variance in timing through software, hardware and internet connection speed—and still be robust enough to detect RT differences, even in a task containing a relatively low number of trials (< 100).

Results such as these provide evidence that could enable more researchers to undertake behavioral research on the web, whilst also offering the maintained back end that can be kept up to date with changes in user’s browsers—that otherwise would require a much higher level of technical involvement.

Building on these advantages, Gorilla is currently being used to teach research methods to undergraduate students in London at University College London and Birkbeck, University of London. In comparison with other software, requiring specific programming skills, the teaching teams noted a lower need to provide technical assistance to students, allowing them to better focus on research design per se.

### Limitations

While technical involvement is lowered with Gorilla, there are still some limitations with presenting a task in a browser that the user should be aware of. These are mainly limited to timing issues, which Gorilla minimizes but does not eliminate—there will always be room for timing errors, even though it is decreased. The specific reasons for these errors, and how they may be quantified or overcome in the future, are discussed below.

As with any software running on a user’s device, Gorilla’s response time is limited by the sampling/polling rate of input devices—a keyboard, for example. Unfortunately, short of installing intrusive software on the user’s device, the web browser has no mechanism for directly accessing polling rate—or controlling for polling rate. Often this sits at around 125 Hz, so this can be used to inform conclusions based on RT data gathered online. Future developments may at some point allow programs running in the browser to access hardware information and adjust for this—however, this will only be important for research that aims to model individual trials on an accuracy of less than 8 ms (the default USB polling rate for input devices is 125 Hz, so a sample every 8 ms). Alternatively, developments in recruitment platforms (such as Prolific and Mechanical Turk) may enable screening of participant’s hardware, allowing researchers to specify participants with high refresh monitors and high-polling-rate input devices (most likely to be video gamers). This would reduce the online research benefit of a larger available participant pool, but there are still many large and diverse groups of participants who meet such requirements, including the PC gaming community. Online research specifically targeting the gaming community has successfully gathered large amounts of data in the past (Ipeirotis & Paritosh, 2011; Ross, Irani, Silberman, Zaldivar, & Tomlinson, 2010).

One unique problem in remote testing is the potential processing load any given participant may have running on their computer may vary dramatically. High processing loads will impact the consistency of stimulus presentation and the recording of responses. Fortunately, the platform records the actual time each frame is presented for, against the desired time—so the impact on timing can be recorded and monitored. A potential future tool would be a processing load check—this could either work by performing computations in the browser and timing them as a proxy for load. Or, it may potentially become possible to measure this using methods already available in Node.js (an off-browser JavaScript runtime engine) for profiling CPU performance—something that is likely to become possible if performance.now() timing is—at least partially—reinstated in browsers (for examples of how this could work, see Nakibly, Shelef, & Yudilevich, 2015; Saito et al., 2016).

The use of modern browser features, such as requestAnimationFrame(), gives the best possible timing fidelity in the browser environment, and also allows for inconsistencies in frame refresh rates to be measured and accounted for. Online research will always be limited by the hardware that participants have, and despite the availability of modern monitors offering higher frame rates, most users’ systems operate a refresh rate of 60 Hz (Nakibly et al., 2015; Zotos & Herpers, 2012, 2013), therefore most stimulus presentation times are limited to multiples of 16.667 ms. Giving some insight into online participant’s device usage, a Mechanical Turk survey showed that over 60% of users were using a laptop, phone, or tablet—the vast majority of which have a 60-Hz refresh rate (Jacques & Kristensson, 2017). It is therefore advisable for users on any online platform to restrict presentation times to multiples of 16.667 ms. This is spoken about in Gorilla’s documentation; however, a future feature might be to include a warning to users when they try to enter nonmultiples of the standard frame rate.

### New and Future features

Some potential improvements to the platform would make it a more powerful tool for researchers. These fall into two camps: tools for widening the range of experiments you can run, and tools for improving the quality of data you can collect.

In the authors’ experience, tools for researchers to run online visual perception, attention and cognition research are limited. This is perhaps a product of reluctance to use online methods, due to concerns regarding timing—which we hope to have moved toward addressing. To provide a greater range of tools a JavaScript-based Gabor patch generator has been developed, which can be viewed using this link: www.bit.ly/GorillaGabor documentation for this tool is avaible at: www.gorilla.sc/support/reference/task-builder-zones#gabor-patch. This first asks participants to calibrate their presentation size to a credit card, and measure the distance to the screen—calculating visual degrees per pixel—and then allows presentation of a Gabor patch with size, frequency, window size in degrees. Experimenters can also set animations that change the phase and angle of these patches over time. These animations are fast (40 Hz), because the patch and window are pregenerated and manipulated to produce the animation, rather than a frame-by-frame new patch generation.

Another tool that widens online research capabilities is remote, webcam-based eyetracking. An implementation of the WebGazer.js library (Papoutsaki et al., 2016) for eyetracking has also been integrated into the platform. This permits rough eyetracking, and head position tracking, using the user’s webcam. Recent research has provided evidence that this can be used for behavioral research, with reasonable accuracy—about 18% of screen size (Semmelmann & Weigelt, 2018). This also includes a calibration tool, which can be run as frequently as needed, which allows for the quantification of eyetracking accuracy, and offers the ability to end the experiment if the webcam cannot be calibrated to the desired level. A demo of the calibration is available here: www.bit.ly/EyeDemo, and documentation is available at www.gorilla.sc/support/reference/task-builder-zones#eye-tracking. Additionally, WebGazer.js allows the experimenter to track the presence and changes in distance, of a user’s face. This can help with data quality, as you can assess when a user is looking at the screen, and prompt them to remain attentive to the task. The impact of this type of monitoring may be particularly interesting to investigate in a task such as the one presented in this article—perhaps participants would show a different flanker effect if they were more attentive in the task.

Another feature Gorilla has introduced is Gorilla Open Materials, which is an open-access repository where experiments, tasks and questionnaires can be published. This will enable other users to: experience study protocols, inspect the configuration settings of tasks and questionnaires, and clone study protocol, tasks and questionnaires for their own research. This increases the transparency, accessibility and reproducibility of published research. As the repository grows, we hope it will also allow researchers to build on what has gone before without needing to reinvent the wheel. A summary is availble here: www.gorilla.sc/open-materials.

### Conclusion

We have described Gorilla as a tool that significantly lowers the access barriers to running online experiments—for instance, understanding web development languages, servers, and programming APIs—by managing all levels of implementation for the user and keeping up to date with changes in the browser ecosystem. We presented a case study, to demonstrate Gorilla’s capacity to be robust to environmental variance (from software, hardware, and setting) during a timing task. An RT-sensitive flanker effect—Rueda et al.’s (2004) “conflict network”—was replicated in several populations and situations. Some constraints in running studies online remain, but there may be future ways of tackling some of these (i.e., with specialist hardware). Future improvements to the platform will include a Gabor generator, webcam eyetracking, and movement monitoring.

## Electronic supplementary material

ESM 1 (PDF 517 kb)

## Footnotes

1 Note that recent developments in web browsers have introduced different application programming interfaces (APIs)—for example, the audio API in Chrome—that allow access to audio devices; calls can be made to these APIs from JavaScript that are outside the event loop and are executed asynchronously. For a list of examples, see www.developer.mozilla.org/en-US/docs/Web/API.
