# Supplementary material for: Gorilla in our midst: An online behavioral experiment builder
Source: Behav Res Methods. 2019 Apr 23;52(1):388–407. doi: 10.3758/s13428-019-01237-x (PMC7005094; doi:10.3758/s13428-019-01237-x)
Supplement: Supplementary file 1 — (PDF 517 kb) [file 13428_2019_1237_MOESM1_ESM.pdf]

Supplementary Material: A tutorial on constructing the Flanker task

The Flanker task was built in two steps. First, we created the Task. Secondly, we incorporated this Task in an Experiment, which allowed us to recruit participants.

Creating the task

The Flanker task is a fairly simple task to program on Gorilla. Out of the five tabs of the Task Builder (Task Structure, Spreadsheet, Stimuli, Manipulations, Script), we only need to use to first three ones.

The "Task Structure" (highlighted in red below) tab allows us to define the different sections within the task, and the different screens within each section.

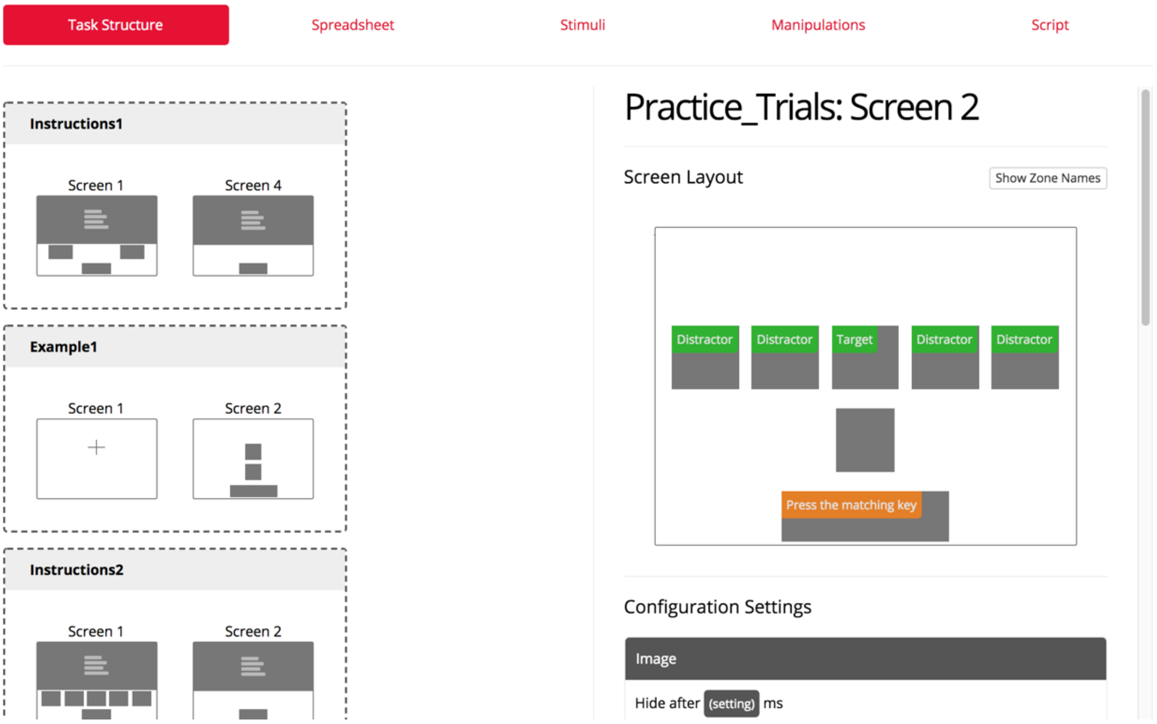

On the left, we can see the different sections. There is a first set of instructions ("Instructions 1"), followed by a very simple demonstration of the stimuli ("Example 1"). The instructions are fully developed in "Instructions 2" and followed by some practice trials ("Practice_Trials").

On the right, we can see more specifically how the screen is designed for the practice trials. Five images zones have been defined. Their name is written in green: the central fish is the "Target", the flanker fish the "Distractors". The rectangular zone with an orange label indicates the response modality. Participants have to press a button saying whether the central fish is pointing to the right ("m") or to the left ("z"). They will see the instructions that are written in orange on the screen: "Press the matching key". The squared zone in between the green and orange zones is used to provide feedback. We will not develop it here since it is only used during practice, and not during the main task.

Crucially, the Screen set up only has to be defined once. The images that will populate it from trial to trial are specified in the Spreadsheet.

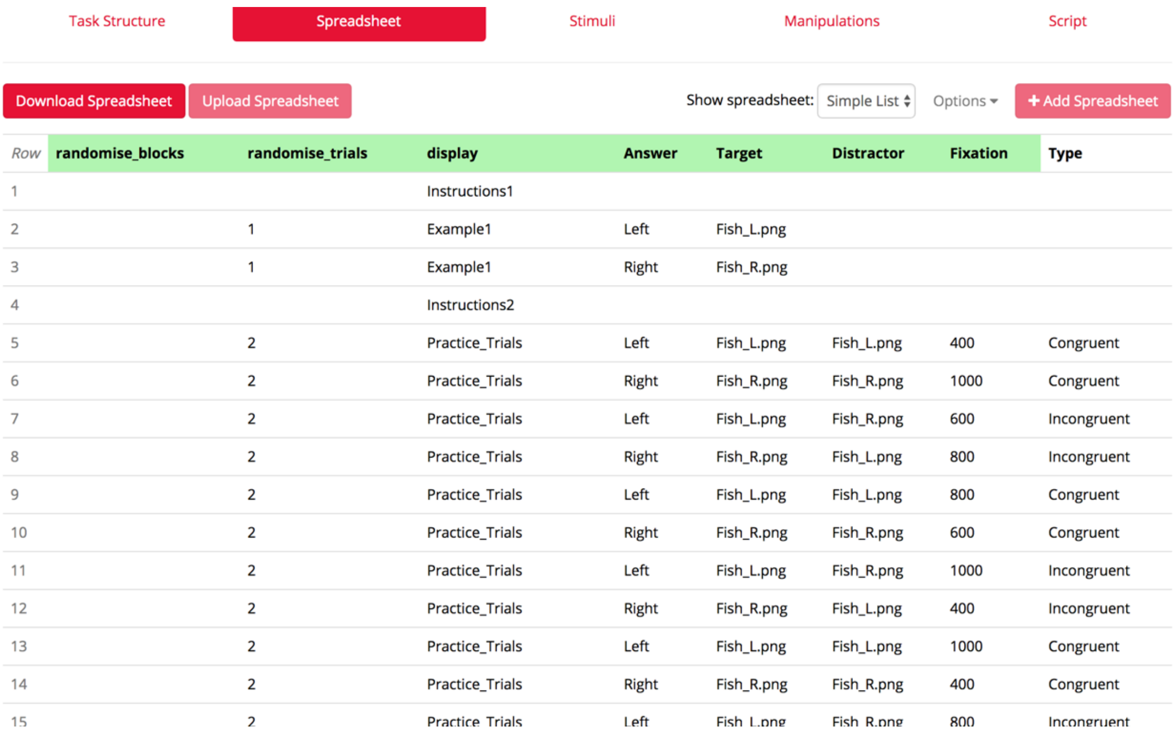

Row	randomise_blocks	randomise_trials	display	Answer	Target	Distractor	Fixation	Type
1			Instructions1					
2		1	Example1	Left	Fish_L.png			
3		1	Example1	Right	Fish_R.png			
4			Instructions2					
5		2	Practice_Trials	Left	Fish_L.png	Fish_L.png	400	Congruent
6		2	Practice_Trials	Right	Fish_R.png	Fish_R.png	1000	Congruent
7		2	Practice_Trials	Left	Fish_L.png	Fish_R.png	600	Incongruent
8		2	Practice_Trials	Right	Fish_R.png	Fish_L.png	800	Incongruent
9		2	Practice_Trials	Left	Fish_L.png	Fish_L.png	800	Congruent
10		2	Practice_Trials	Right	Fish_R.png	Fish_R.png	600	Congruent
11		2	Practice_Trials	Left	Fish_L.png	Fish_R.png	1000	Incongruent
12		2	Practice_Trials	Right	Fish_R.png	Fish_L.png	400	Incongruent
13		2	Practice_Trials	Left	Fish_L.png	Fish_L.png	1000	Congruent
14		2	Practice_Trials	Right	Fish_R.png	Fish_R.png	400	Congruent
15		2	Practice_Trials	Left	Fish_L.png	Fish_R.png	800	Incongruent

The "Display" columns mirrors the sections that have been created in our "Task Structure". Let's focus on the "Practice_Trials" display. In the "Target" and "Distractor" columns, we indicate which image will be uploaded for each trial. On the first Practice Trial, for example (Row 5), the Target is a fish pointing to the left, and the Distractors are also pointing to the left: it is actually a Congruent trial, as written in the Column "Type". The images for each zone (here it is only "Fish_L.png") have to be

uploaded in the “Stimuli” menu.

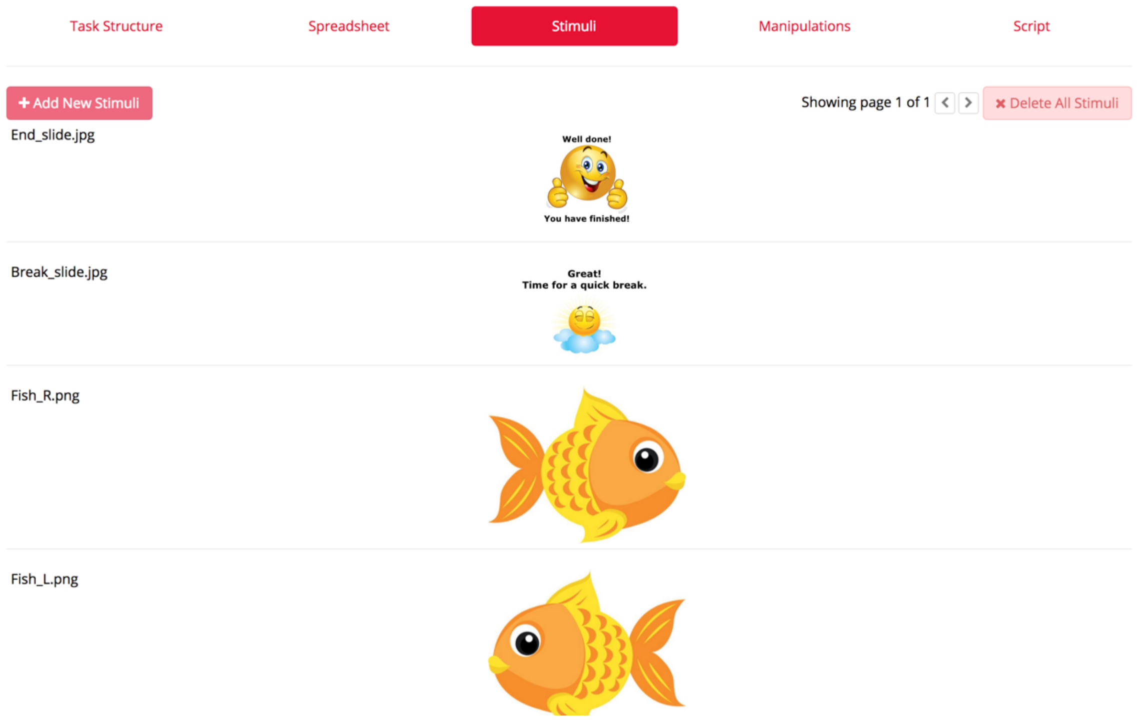

The last image is the one that is called “Fish_L.png”. Once we have set up our task, using the “Task structure”, the “Spreadsheet” and the “Stimuli” tabs, we need to put it in an experiment.

Putting the task into an experiment

Gorilla uses what we call an “Experiment tree”. The tree specifies which tasks are used within an experiment, as well as their order. Here, the experiment (Called “Flanker Fish”) is pretty simple, because we only use the Flanker Task.

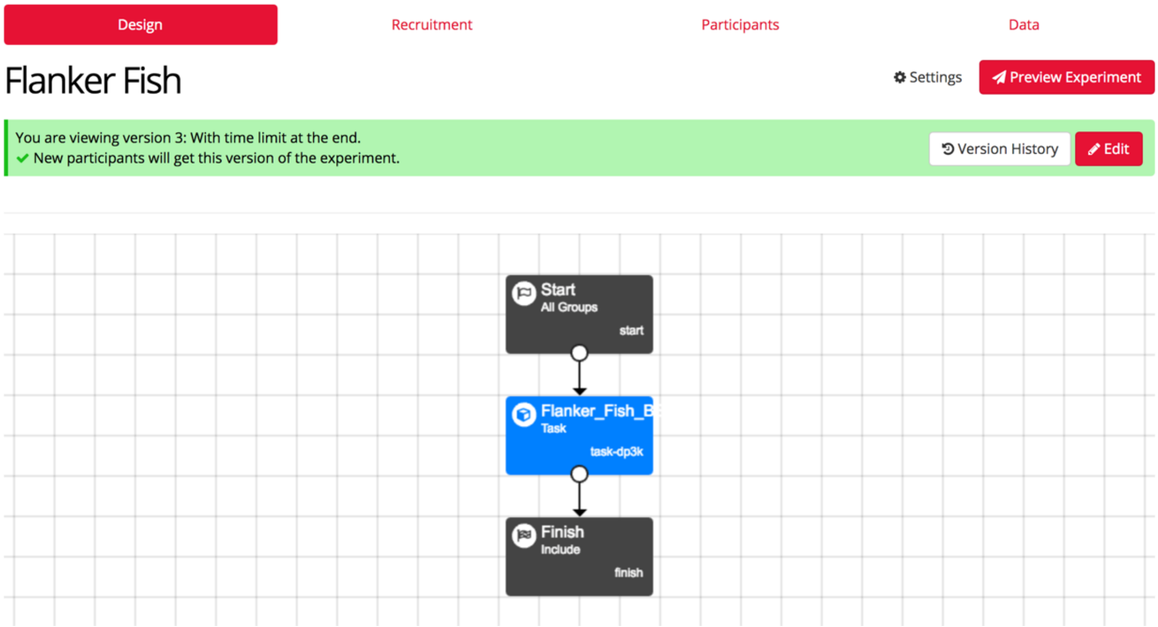

It is shown in blue, and it is surrounded by a "Start node", and a "Finish node", specifying the beginning and the end of the experiment. Many more functionalities are available in the "Experiment tree", allowing researchers to counterbalance the order of presentation for an experiment that contains several tasks, for example.

The "Recruitment tab" allows us to generate a link to share the experiment online, and to select how many participants we would like to recruit, along with any specific technical requirement (device types, browser types of connection speed that the participants would need to have). The "Participants" tab references all the participants that joined the experiment, and the "Data" tab allows us to download our data.
